# The prognosis analysis of organ metastatic patterns in lung large cell neuroendocrine carcinoma: A population-based study

**DOI:** 10.3389/fonc.2022.1050800

**Published:** 2022-12-08

**Authors:** Kai Chen, Peiling Dai, Jiangwei Ni, Yili Xiang, Lizhong Gu

**Affiliations:** ^1^ The Department of Cardiovascular and Thoracic Surgery, The Second Affiliated Hospital of Wenzhou Medical University, Wenzhou, China; ^2^ The Department of Radiotherapy, The First Affiliated Hospital of Wenzhou Medical University, Wenzhou, China; ^3^ The Department of Thoracic Surgery, The First Affiliated Hospital of Wenzhou Medical University, Wenzhou, China; ^4^ Department of Interventional Vascular Surgery, Wenzhou People’s Hospital, The Third Affiliated Hospital of Shanghai University, Wenzhou, Zhejiang, China

**Keywords:** lung large cell neuroendocrine carcinoma, prognosis, metastatic patterns, overall survival, SEER

## Abstract

Lung large cell neuroendocrine carcinoma (LCNEC) is a rare and highly aggressive malignancy with a dismal prognosis. This study was designed to depict patterns of distant organ metastatic and to analyze prognosis of LCNEC patients. We gathered data from the Surveillance, Epidemiology, and End Results (SEER) database between 2010 and 2015. We conducted the Kaplan–Meier method to calculate overall survival (OS) and compare different variables. Cox proportional hazards regression models in univariate and multivariate analyses were employed to further explore prognostic factors. A total of 1335 LCNEC patients were eventually selected from the SEER database, of which 348 patients (26.0%) had single organ metastasis and 197 patients (14.8%) had multiple metastases. Our study indicates that patients with single organ metastasis generally have a poor prognosis, with a median OS of 8 months for both lung and brain metastasis with 1-year survival rates of 33% and 29% respectively. Patients with multiple metastases exhibited the worst prognosis, with a median OS of only 4 months and a 1-year OS of 8%. Multivariate analysis revealed that age, T stage, N stage, chemotherapy and radiation in metastatic patients were independently associated with OS. In conclusion, LCNEC exhibits a high metastatic rate when diagnosed. The most common metastatic organ is the brain in single-site metastatic patients. Patients with single or multiple metastases exhibit a significantly worse prognosis than those with non-organ metastases. In the group of single organ metastases, patients with brain and lung metastases had a better prognosis than those with bone and liver metastases.

## Introduction

Lung large cell neuroendocrine carcinoma (LCNEC) of the lung is a rare, malignant tumor with poor prognosis and a high recurrence rate (1). Previous studies have suggested that the incidence of LCNEC is low, at a rate of 3% of all lung cancers (2). However, studies have shown that the incidence of LCNEC is rising (3, 4). According to the World Health Organization (WHO) classification of lung tumors in 2015, LCNEC was removed from large cell carcinoma, which is a subtype of non-small cell lung carcinoma (NSCLC), and recognized as a pulmonary neuroendocrine tumor (NET) together with typical carcinoid (TC), atypical carcinoid (AC) and small-cell lung carcinoma (SCLC) (5). Varlotto et al. reported that LCNEC exhibited clinicopathological features and prognoses more similar to NSCLC than SCLC (3). However, other studies have suggested that LCNEC shares several clinicopathological characteristics with SCLC, including a high degree of aggressiveness, poor prognosis, smoking-related disease, genetic mutations and neuroendocrine gene expression (1, 6–9).

Additionally, 65% of LCNEC patients are metastatic when diagnosed (10). Most literature reports that the most common metastatic site of SCLC is the liver, and the most common site of NSCLC is bone (11, 12). However, few studies have focused on the patterns of distant site metastasis in LCNEC. It is well known that the metastatic spread of distant organs in cancer is a significant factor affecting the prognosis of patients. Generally, stage IV LCNEC presents with extensive metastatic disease and has poor survival outcomes (<10 months), similar to SCLC (9, 13). Unfortunately, the correlation between specific-site metastatic patterns and prognosis in LCNEC remains unclear.

This study was conducted using patient data extracted from the Surveillance Epidemiology and End Results (SEER) database from 2010 to 2015. Our objectives are to delineate the patterns of distant organ metastasis and to investigate the survival outcomes of metastatic LCNEC patients and significant factors impacting prognosis, which could offer pivotal evidence for pre-evaluation and optimal follow-up strategy selection.

## Materials and methods

### Database source

The SEER database is the largest publicly available cancer database, which collects information on cancer incidence and prognosis from 17 population-based cancer registries. This database covers approximately 30% of the American population. It is one of the most representative large clinical cancer registration databases in North America. It provides useful first-hand information of cancers for doctors. Since data from SEER are publicly available and de-identified, this study was exempt from local institutional review board review. SEER Stat software (SEER Stat 8.3.5) was used to extract the data.

### Patient selection

**Figure 1:**
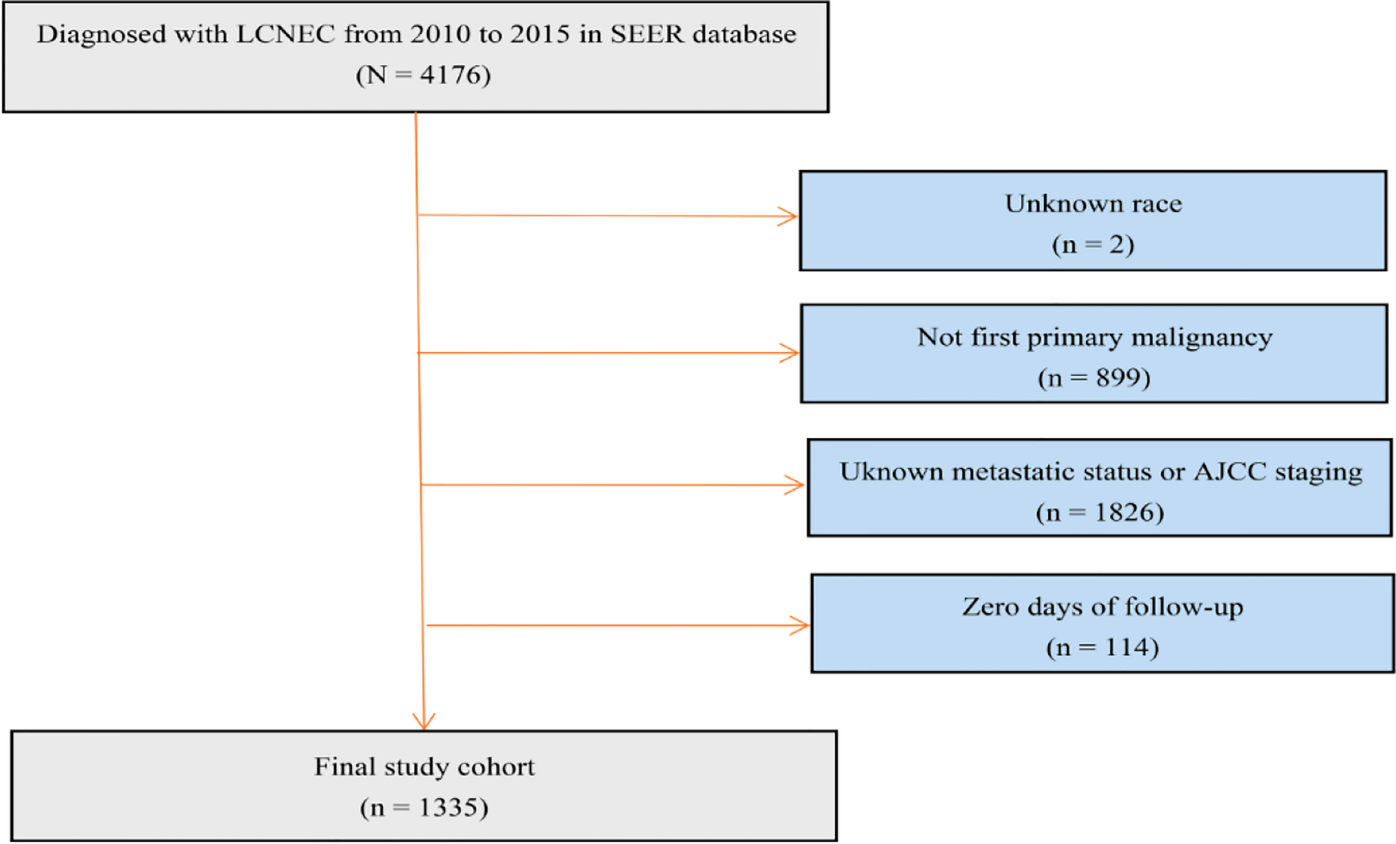
Flow chart of patient cohort selection. LCNEC, large cell neuroendocrine carcinoma.

We established a cohort study using data extracted from the SEER database from 2010 to 2015. ICD – O - 3 (International Classification of Diseases for Oncology, 3rd edition) morphology codes 8013/3 were used to identify LCNEC. The detailed cohort selection procedure is summarized in **Figure 1**. In short, we identified 1335 patients diagnosed with LCNEC between 2010 and 2015. Patients were excluded if LCNEC was not the first primary malignancy; if race, metastatic status or AJCC 7th staging were unknown; and if they had no follow-up data.

### Covariates

Covariates included age at diagnosis (≤50 years, >50 years), gender (male, female), race (white, black, other), primary site (main bronchus upper lobe, middle lobe, lower lobe, others), T stage (T0-2, T3-4, Tx), N stage (N0, N1, N2, N3, Nx), AJCC 7th stage (I, II, III, IV), surgery (yes, no/unknown), chemotherapy (yes, no/unknown) and radiation (yes, no/unknown). Information about distant metastatic sites was obtained mainly from the medical records in the SEER program. For the prognosis study, we divided the patients into 6 groups: nonmetastatic; lung metastasis; liver metastasis; bone metastasis; brain metastasis and multiple organ metastasis. Multiple means metastases in at least 2 of the above sites. Other metastases (bone, brain, liver, lung) represent an isolated lesion. The endpoint of this study was OS, which was defined as the time interval from diagnosis to the most recent follow-up date, or date of death. We also calculated the 1-year survival rate for prognostic comparison.

### Statistical analysis

Statistical Product and Service Solutions (SPSS) (26.0, IBM, Armonk, NY, USA) was used for statistical analysis. Metastatic distribution information was provided for bone, brain, liver and lung. OS was estimated using the Kaplan‐Meier method by log-rank test with ggplot2, survminer and survival packages in R software version 3.6.0. Adjusted HRs with 95% CIs were calculated using Cox proportional hazards regression models to estimate prognostic factors associated with OS. P-values < 0.05 were considered statistically significant.

## Results

### Patient characteristics

We finally selected 1335 patients from the SEER database. The baseline characteristics are presented in **Table 1**. Patients included 725 men (54.3%) and 610 women (45.7%). Most of the patients were elderly people: 1235 patients (92.5%) were >50 years old, while 142 patients (7.5%) were ≤50 years old. Only a small number of tumors (4.2%, N = 56) originated from the main bronchus, while more than half of the tumors occurred in the upper lobe of the lung (56.6%, N = 755). A total of 49.1% (N = 656) of patients were in stage IV, while 22.2% (N = 297), 10.3% (N =138) and 18.3% (N = 244) were in stages I, II and III, respectively. Based on the available information, 36.4% of patients (N = 486) underwent surgery. In addition, 770 (57.7%) patients received chemotherapy and 264 (19.8%) patients were treated with radiotherapy.

**Table 1:** Clinicopathological characteristics of patients with metastatic LCNEC.

Characteristics	Bone metastasis(N=73)	Brainmetastasis(N=159)	Liver metastasis(N=68)	Lung metastasis(N=48)	Multiplemetastasis(N=197)	Non-organmetastasis(N=790)	TotalMetastasis(N=1335)
Age, n (%)							
≤50	7 (9.6)	16 (10.1)	5 (7.4)	4 (8.3)	12 (6.1)	56 (7.1)	100 (7.5)
>50	66 (90.4)	143 (89.9)	63 (92.6)	44 (91.7)	185 (93.9)	734 (92.9)	1235 (92.5)
Sex, n (%)							
Male	53 (72.6)	79 (50.0)	36 (52.9)	31 (64.6)	118 (59.9)	408 (51.6)	725 (54.3)
Female	20 (27.4)	80 (50.0)	32 (47.1)	17 (35.4)	79 (40.1)	382 (48.4)	610 (45.7)
Race, n (%)							
White	57 (78.1)	134 (84.3)	61 (89.7)	35 (72.9)	169 (85.8)	651 (82.4)	1107 (82.9)
Black	12 (16.4)	21 (13.2)	4 (5.9)	9 (18.8)	20 (10.2)	111 (14.1)	177 (13.3)
Others	4 (5.5)	4 (2.5)	3 (4.4)	4 (8.3)	8 (4.1)	28 (3.5)	51 (3.8)
Primary Site, n (%)							
Main bronchus	6 (8.2)	6 (3.8)	9 (13.2)	4 (8.3)	17 (8.6)	14 (1.8)	56 (4.2)
Upper lobe	39 (53.4)	81 (50.9)	29 (42.6)	23 (47.9)	87 (44.2)	496 (62.8)	755 (56.6)
Middle lobe	1 (1.4)	5 (3.1)	8 (11.8)	0 (0)	10 (5.1)	29 (3.7)	53 (4.0)
Lower lobe	15 (20.5)	38 (23.9)	14 (20.6)	13 (27.1)	50 (25.4)	194 (24.6)	324 (24.3)
Others	12 (16.4)	29 (18.2)	8 (11.8)	8 (16.7)	33 (16.8)	57 (7.2)	147 (11.0)
T stage, n (%)							
T0-2	25 (34.2)	66 (41.5)	30 (44.1)	13 (27.1)	68 (34.5)	522 (66.1)	724 (54.2)
T3-4	41 (56.2)	69 (43.4)	25 (36.8)	33 (68.8)	107 (54.3)	242 (30.6)	517 (38.7)
TX	7 (9.6)	24 (15.1)	13 (19.1)	2 (4.2)	22 (11.2)	26 (3.3)	94 (7.0)
N stage, n (%)							
N0	14 (19.2)	60 (37.7)	12 (17.6)	11 (22.9)	26 (13.2)	436 (55.2)	559 (41.9)
N1	5 (6.8)	15 (9.4)	7 (10.3)	2 (4.2)	20 (10.2)	81 (10.3)	130 (9.7)
N2	29 (39.7)	63 (39.6)	33 (48.5)	20 (41.7)	92 (46.7)	198 (25.1)	435 (32.6)
N3	2027.4)	16 (10.1)	13 (19.1)	15 (31.2)	50 (25.4)	72 (9.1)	186 (13.9)
NX	5 (6.8)	5 (3.1)	3 (4.4)	0 (0)	9 (4.6)	3 (0.4)	25 (1.9)
AJCC 7th Stage, n (%)							
I	–	–	–	–	–	297 (37.6)	297 (22.2)
II	–	–	–	–	–	138 (17.5)	138 (10.3)
III	–	–	–	–	–	244 (30.9)	244 (18.3)
IV	73 (100)	159 (100)	68 (100)	48 (100)	197 (100)	111 (14.1)	656 (49.1)
Surgery, n (%)							
Yes	4 (5.5)	16 (10.1)	2 (2.9)	5 (10.4)	8 (4.1)	451 (57.1)	486 (36.4)
No/Unknown	69 (94.5)	143 (89.9)	66 (97.1)	43 (89.6)	189 (95.9)	339 (42.9)	849 (63.6)
Radiation, n (%)							
Yes	19 (26.0)	80 (50.0)	4 (5.9)	3 (6.3)	35 (17.8)	123 (15.6)	264 (19.8)
No/Unknown	54 (74.0)	79 (50.0)	64 (94.1)	45 (93.8)	162 (82.2)	667 (84.4)	1071 (80.2)
Chemotherapy, n (%)							
Yes	52 (71.2)	97 (61.0)	43 (63.2)	35 (72.9)	139 (70.6)	404 (51.1)	770 (57.7)
No/Unknown	21 (28.8)	62 (39.0)	25 (36.8)	13 (27.1)	58 (29.4)	386 (48.9)	565 (42.3)

Multiple mean metastases in at least 2 of the above sites. Other metastases (bone, brain, liver, lung) represent isolated lesion. LCNEC, large cell neuroendocrine carcinoma.

**Figure 2:**
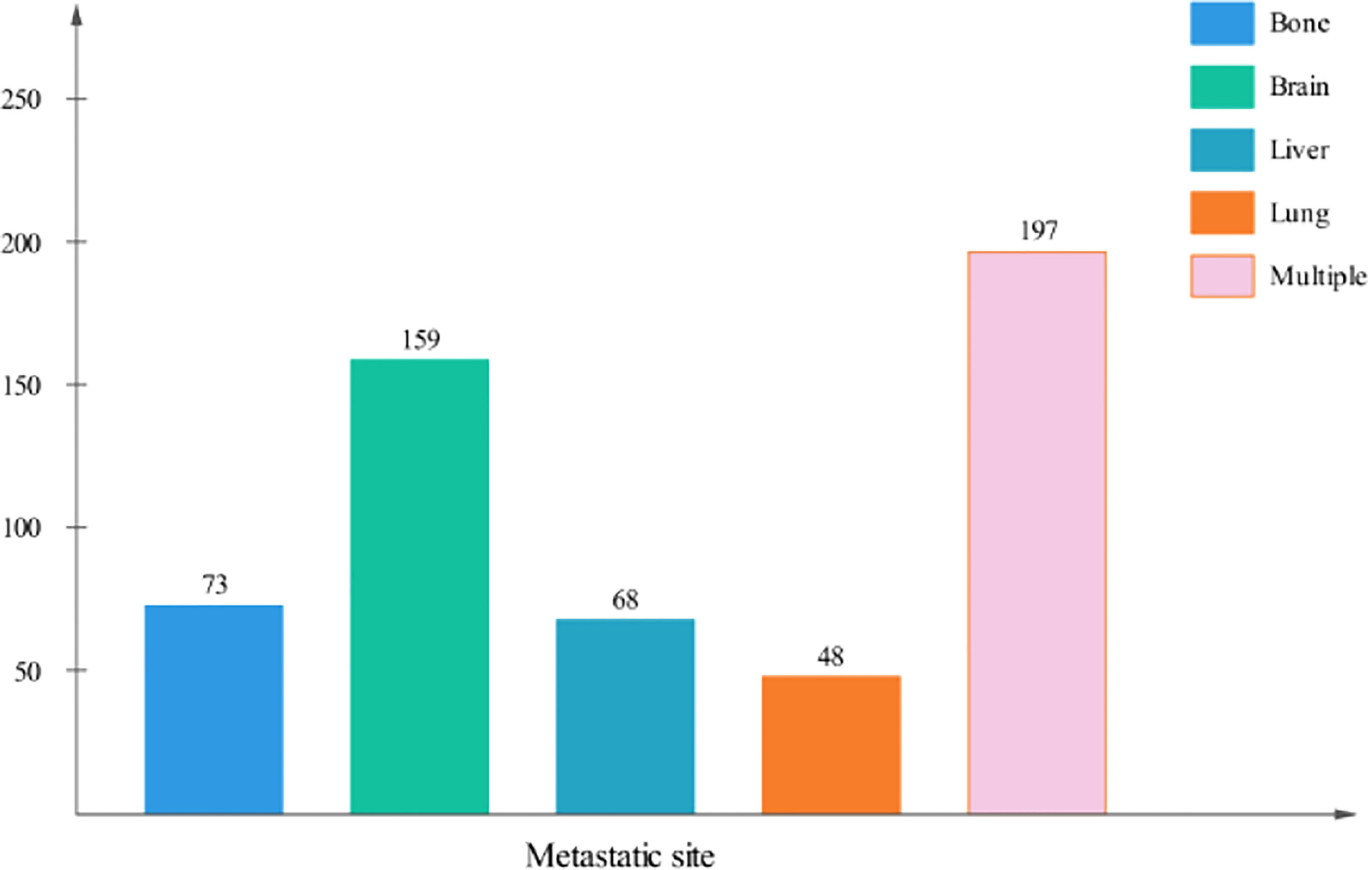
Number of patients with different metastatic patterns in LCNEC.

The metastatic patterns of distant organs are shown in **Figure 2**. A total of 790(59.2%) LCNEC patients had no organ metastasis. A total of 348(26.0%) patients had a single organ site of metastasis, while 197 patients (14.8%) had multiple organ metastases. Of the patients with a single metastasis, 159 (45.7%) patients were diagnosed with isolated brain metastasis, 73 (21.0%) patients were diagnosed with isolated liver metastasis, 68 (19.5%) patients were diagnosed with isolated bone metastasis and 48 (13.8%) patients were diagnosed with isolated lung metastasis.

### Survival outcomes

**Figure 3:**
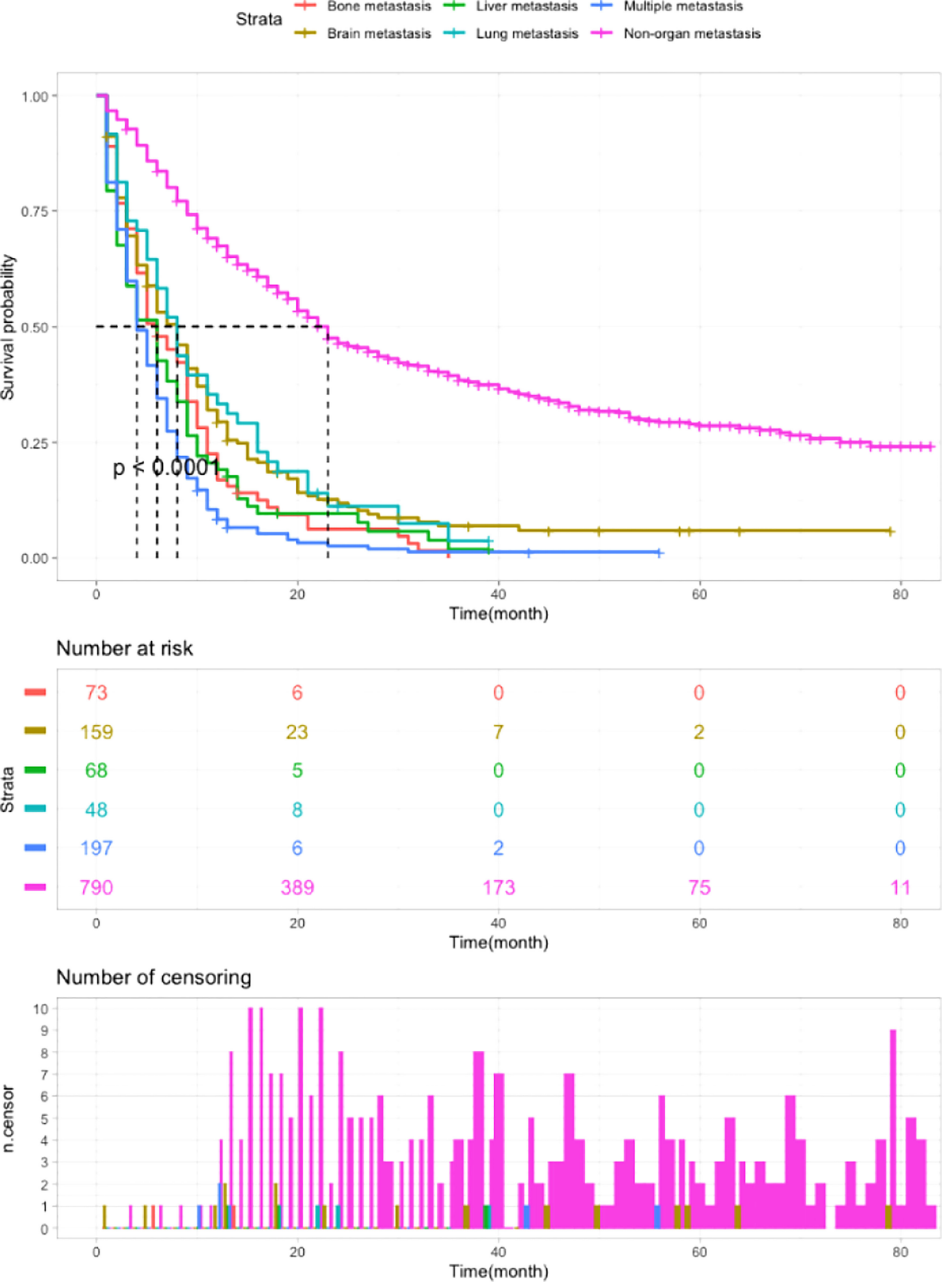
Kaplan-Meier analysis of the OS of LCNEC patients with different metastatic sites (P < 0.0001).

As shown in **Table 2**, survival analysis was performed to determine the OS of patients with different metastatic patterns. Patients without organ metastasis had the best survival outcomes, with a median OS of 23 months and a 1-year OS of 67%. The median OS of patients with multiple metastases was 4 months and the 1-year OS was 8%, which indicates the worst outcomes in metastatic patients. For patients with single site metastasis, those with bone and liver metastases had poorer survival than those with brain and lung metastases. (bone metastasis: median OS=6m, 1-year OS=17%; liver metastasis: median OS=6m, 1-year OS=19%; lung metastasis: median OS=8m, 1-year OS=33%; brain metastasis: median OS=8m, 1-year OS=29%; multiple metastases: median OS=4m, 1-year OS=8%. non-organ metastasis *vs* other groups: P<0.0001). **Figure 3** exhibits the survival curves generated by Kaplan-Meier analysis, which confirmed the above results.

**Table 2:** Survival analysis of various metastatic organs.

Parameter	1-year OS (%)	Median OS (months)
Bone metastasis	17	6.0
Brain metastasis	29	8.0
Liver metastasis	19	6.0
Lung metastasis	33	8.0
Multiple metastasis	8	4.0
Non-organ metastasis	67	23.0

**Figure 4:**
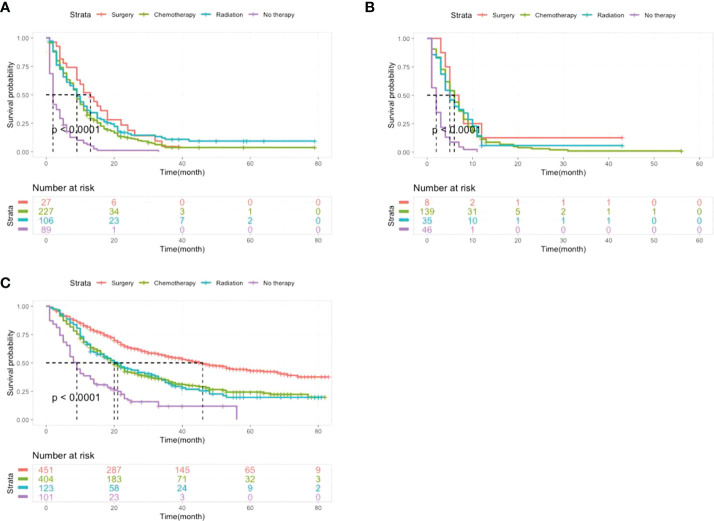
Kaplan-Meier analysis of the OS of LCNEC patients with different metastatic sites receiving different treatments. **(A)** Patients with single organ metastasis. **(B)** Patients with multiple organ metastasis. **(C)** Patients without organ metastasis.

As shown in **Figure 4**, for the group without metastasis, patients with intervention had better outcomes for OS than patients without therapy (P<0.0001), and surgery had significantly better outcomes than chemotherapy and radiation (P<0.0001). Among patients with one and multiple metastases, patients who received surgery, chemotherapy or radiotherapy had a better prognosis than those who did not receive therapy (P<0.0001), however, there were no significant differences between the interventions (P=0.23).

### Univariate and multivariate survival analysis of metastatic patients

Univariate analysis suggested that age, T stage, N stage, surgery, radiotherapy, and chemotherapy had significant impacts on OS, as shown in **Table 3**. The results of multivariate analysis indicated that age, T stage, N stage, radiation and chemotherapy were all significantly correlated with OS. Specifically, patients ≤50 years old had a better OS than patients >50 years old (HR: 0.60, 95% CI: 0.43–0.84, P<0.01). Patients receiving chemotherapy and radiation had a better prognosis (HR: 0.66, 95% CI: 0.53–0.82, P<0.01; HR:0.44, 95% CI:0.36–0.53, P<0.01). Different T stages and N stages had significant impacts on prognosis (P=0.01, P<0.01).

**Table 3:** Univariate and multivariate Cox regression analyses of prognostic factors in the overall LCNEC metastatic patient cohort.

Characteristics	Univariate analysis			Multivariate analysis		
	HR	95% CI	P-value	HR	95% CI	P-value
Age						
≤50	1.00			1.00		
>50	0.59	0.42-0.82	< 0.01	0.60	0.43-0.84	< 0.01
Sex				NA		
Male	1.00					
Female	1.04	0.88-1.24	0.64			
Race			0.34	NA		
White	1.00					
Black	1.06	0.69-1.64	0.77			
Others	0.87	0.54-1.42	0.58			
Primary Site			0.55	NA		
Main bronchus	1.00					
Upper lobe	1.35	0.93-1.97	0.12			
Middle lobe	1.07	0.83-1.38	0.59			
Lower lobe	1.06	0.67-1.66	0.81			
Others	1.16	0.88-1.54	0.29			
T stage			0.01			0.01
T0‐2	1.00			1.00		
T3‐4	0.94	0.70-1.26	0.68	1.08	0.79-1.47	0.63
TX	1.26	0.95-1.66	0.11	1.39	1.04-1.87	0.03
N stage			< 0.01			<0.01
N0	1.00			1.00		
N1	0.61	0.38-0.97	0.04	0.69	0.42-1.13	0.14
N2	0.80	0.48-1.34	0.40	0.89	0.52-1.52	0.67
N3	0.93	0.60-1.46	0.76	1.11	0.70-1.78	0.66
NX	0.96	0.60-1.53	0.86	1.16	0.71-1.89	0.56
Surgery						
Yes	1.00			1.00		
No/Unknown	0.57	0.40-0.83	< 0.01	0.70	0.47-1.02	0.06
Radiation						
Yes	1.00			1.00		
No/Unknown	0.62	0.51-0.77	< 0.01	0.66	0.53-0.82	< 0.01
Chemotherapy						
Yes	1.00			1.00		
No/Unknown	0.52	0.43-0.63	< 0.01	0.44	0.36-0.53	< 0.01

LCNEC, large cell neuroendocrine carcinoma; NA, not available.

## Discussion

LCNEC is a rare, malignant pulmonary tumor, and most LCNEC studies are small series due to its rarity. Therefore, the clinicopathological features and prognosis of this entity remain unclear, and few have studied the prognosis of different LCNEC metastatic patterns. We conducted a population-based retrospective study to investigate the survival outcomes of metastatic patterns and treatment methods. Our study indicates that LCNEC patients have a high rate of distant organ metastasis, and the most common metastatic site is the brain. Compared with patients with single or multiple site metastases, patients with non-organ metastasis had the best OS, while the prognosis of patients with metastasis was markedly worse. For patients with one-site metastasis, the prognosis of patients with lung and brain metastasis patients was better than that of patients with liver and bone metastasis. The finding suggests that the type and number of metastatic organs may affect the prognosis of patients with LCNEC. Multivariate analysis showed that age, T stage, N stage, radiotherapy and chemotherapy were independent prognostic factors. Our results on the patterns of distant site metastasis and the prognostic differences of metastatic patients can help to assess patients before treatment and guide the selection of treatment methods.

Cai et al. (14) reported that the most common isolated metastatic site in LCNEC was the brain, while the bone and liver were the most common metastatic organs for NSCLC and SCLC, respectively, and the least common sites were the lung, liver, and lung, respectively. However, a paper using data based on the Netherlands Cancer Registry reported that liver metastasis in LCNEC was 47%, followed by metastasis to the bone (32%), brain (23%), and lung (14%) (9). Our results of brain and bone metastasis in single organ metastasis patients were 45.7% and 21%, respectively, which means that the brain is the most common metastatic distant organ. Given that we did not count patients with brain metastases in multiple metastases, the difference from other studies is understandable. As reported, the 5-year OS rate of LCNEC is 13% to 57% (2, 6, 15–19), and the median OS of stage I–II, III and IV LCNEC patients was 32.4, 12.6 and 4.0 months, respectively (9). Our results are similar: the 1-year OS ranged from 67% in the non-organ metastasis group to 8% in the multiple metastasis group, and the median OS ranged from 23 months to 4 months. Among patients with a single metastasis, the prognosis of patients with liver and bone metastasis was worse than that of patients with lung and brain metastasis. These results indicate that the type and number of distant organ metastases can have an impact on prognosis.

As LCNEC is a very rare disease, only few data are available. Therefore, the treatment guidelines have not been established (1, 20). Primary surgery should be the first option in operable patients (TNM stages I and II). Fasano reported that surgery may be curative in approximately 30% of patients; as a result, the optimization of perioperative treatments could offer better prognosis. In one study, early-stage LCNEC patients who underwent resection had a median OS of 48 months (P = 0.000) (21). Our findings are that in patients without metastases, both single and multiple, interventions can improve prognosis over no therapy. Surgery has better outcomes than radiotherapy and chemotherapy in patients without metastasis, while in metastatic patients, the effects of surgery are similar to those of chemotherapy and radiation. Multivariate analysis found that surgery is not a protective factor for OS in metastatic patients, which may be because half of the patients without organ metastasis underwent surgery, while only a small portion of metastatic patients underwent surgery. A prospective study confirmed that LCNEC patients receiving cisplatin-based chemotherapy had RRs and OS rates comparable to those of SCLC, either receiving cisplatin–etoposide (22, 23) or cisplatin–irinotecan (22, 24–27). Moreover, a retrospective study by Prelaj et al. reported that the application of thoracic radiotherapy (TRT) and prophylactic cranial irradiation (PCI) can improve the survival of advanced patients (28), but it lacks statistical significance. Our results confirm that both chemotherapy and radiotherapy can benefit patients.

This study has several major limitations. First, the analyses of the present study were conducted based on the SEER database. As some data was unavailable from the database, the prognostic analyses cannot be performed perfectly. Moreover, the data are retrospective, so there is bias in patient selection methods. This will limit the representativeness of the analysis results. Finally, relevant data of more distal metastases, such as in the adrenal gland and gastrointestinal tract, and the sequence of metastatic sites cannot be determined. The results obtained in the current study need further examination by future well-designed studies to validate the study’s results.

## Conclusion

In conclusion, based on data from the SEER database, LCNEC is a rare lung cancer subtype with poor prognosis. In patients with a single metastasis, brain metastasis was the most common. The site and number of distant metastases were independent prognostic factors for OS in patients with LCNEC. Patients with lung and brain metastasis have better survival outcomes than those with liver and bone metastasis. Patients with multiple metastases have the worst prognosis. Surgery has a significant effect on non-organ metastasis patients. Chemotherapy and radiation therapy benefit most patients. In summary, this study suggests that increased attention should be paid to the patterns and prognostic value of distant metastases for LCNEC.

## Data availability statement

The original contributions presented in the study are included in the article/supplementary material. Further inquiries can be directed to the corresponding author.

## Ethics statement

The studies involving human participants were reviewed and approved by The Second Affiliated Hospital of Wenzhou Medical University. This study is based on human subjects data extracted from the SEER database between 2010 and 2015, thus was exempt from local institutional review board review. Written informed consent for participation was not required for this study in accordance with the national legislation and the institutional requirements.

## Author contributions

KC, PD and LG designed the study and performed the experiments, KC and JN collected the data, PD and YX analyzed the data, KC, PD and LG prepared the manuscript. All authors contributed to the article and approved the submitted version.
